# Physiological Roles and Potential Therapeutic Applications of the P2X7 Receptor in Inflammation and Pain

**DOI:** 10.3390/molecules180910953

**Published:** 2013-09-05

**Authors:** Luiz Anastacio Alves, Rômulo José Soares Bezerra, Robson Xavier Faria, Leonardo Gomes Braga Ferreira, Valber da Silva Frutuoso

**Affiliations:** 1 Laboratory of Cellular Communication, Oswaldo Cruz Foundation, AV. Brazil, 4365, Rio de Janeiro, Brazil; E-Mails: romulo@ioc.fiocruz.br (R.J.S.B); robson.xavier@gmail.com (R.X.F); leonardobraga12@gmail.com (L.G.B.F).; 2 Laboratory of Immnopharmacology, Oswaldo Cruz Foundation, AV. Brazil 4365, Rio de Janeiro, Brazil; E-Mail: frutuoso@ioc.fiocruz.br

**Keywords:** P2X7R, inflammatory response, antagonists and pharmacological target

## Abstract

The P2X7 receptor (P2X7R) is a nonselective cation channel that is activated by extracellular ATP and triggers the secretion of several proinflammatory substances, such as IL-1β, IL-18, TNF-α, and nitric oxide. Recently, several preclinical studies have demonstrated that this receptor participates in inflammation and pain mechanisms. Taken together, these results indicate that P2X7R is a promising pharmacological target, and compounds that modulate the function of this receptor show potential as new anti-inflammatory medicines. In this review, we discuss aspects of P2X7R pharmacology and the participation of this protein in inflammation and pain and provide an overview of some promising compounds that have been tested as antagonists of P2X7R, with clinical applicability.

## 1. Introduction

Inflammation involves many events that attract leukocytes to injured or infected tissue. This homeostatic phenomenon typically leads to tissue repair; however, deregulated inflammation may lead to an overreaction, causing inflammatory disorders. Several components, such as inflammatory mediators (e.g., chemokines, cytokines, and eicosanoids), and biological processes, including cellular activation, migration, adhesion, phagocytosis, and cell death [1], have been implicated in this process. As discussed below, many aspects of the inflammatory reaction are modulated through nucleotides and nucleosides, particularly extracellular adenosine 5′-triphosphate (ATP). Chemotaxis is a key step in the inflammation process. In complex organisms, chemotaxis is defined as a crucial function that is mediated through diverse molecules that attract susceptible cells toward a specific site via concentration gradients. Several diverse chemoattractant substances, such as angiogenic factors, cytokines, hematopoietic growth factors, chemokines, complement components, eicosanoids, and nucleotides and nucleosides [2,3], play particularly important roles in initiating inflammatory responses and recruiting leukocytes to sites of pathogen invasion and/or damaged tissues.

Interestingly, the main chemotaxis purinergic receptor in leukocytes is a G protein-coupled receptor, the P2Y2 receptor. In humans, the receptor P2X1 has been implicated in neutrophil chemokinesis because this receptor increases the chemotactic response to IL-8 [4]. Recently, it has been proposed that the autocrine secretion of ATP is crucial for chemotaxis in macrophages [5].

## 2. The P2X Family and the P2X7 Subtype

Although purinergic signaling was first proposed by Burnstock in 1972 [6], it has taken more than 20 years for most of the scientific community to incorporate the existence of purinergic neurotransmission [7]. There are two major families of purine and pyrimidine receptors: the P1 adenosine receptors (A1, A2a, A2b, and A3), and the P2 (P2R) family, which is divided into the ionotropic (P2X) and metabotropic receptors (P2Y). The ionotropic P2X receptor subfamily comprises seven subtypes of ATP-gated ion channels, called P2X1-7 receptors [8,9]. Among the P2X family, P2X2, P2X4, and certain P2X7 receptors are activated through millimolar concentrations of extracellular ATP, resulting in the opening of a nonselective pore with a 900 Da cut-off in macrophages [9]. In this review, we will focus on the physiological roles and potential therapeutic applications of the P2X7R in inflammation and pain.

## 3. P2X7 Receptor Pharmacology

P2X7R exhibits pharmacological characteristics that differentiate this receptor from other purinergic ionotropic receptors from the family P2 (P2XRs): (1) adenosine 5′-triphosphate (ATP) concentrations greater than 100 µM are required for P2X7R activation; (2) 2,3-O-(4-benzoylbenzoyl)-ATP (BzATP), which is at least 10 times more potent than ATP [10,11,12,13], activates the P2X7R with greater selectivity than P2X1, P2X4, and P2Y11 receptor subtypes [14,15,16]; (3) the effect of ATP is potentiated through a reduction in the extracellular divalent cation concentration [17], although other P2X receptors presented this characteristic [18] and (4) ionic currents show drastic changes in the constant time for closing the channel and amplitude after repeated applications of the same agonist, leading to a non-desensitized state of P2X7R.

Recent findings concerning P2X7R have emphasized several important new considerations about the selectivity and potency of agonist or antagonist molecules *in vitro*. In 2009, Donnelly-Roberts *et al.* [19] showed that BzATP acts on low-conductance ionic channels, such as P2X7R, in humans and exhibits 10 times more potency for inducing ion influx in humans than in rats and mice. Furthermore, *in vitro* experiments using FLIPR (Fluorometric Imaging Plate Reader) assay, BzATP was 10–100 times more efficient at inducing YO-PRO uptake in humans than in both rats and mice. In addition, ATP showed 10 times less activity than BzATP in humans and was a partial agonist at the rat P2X7 receptor [18]. Thus, the cross-reactivity of ATP for the activation of all P2XR subtypes has prompted an intense effort to design selective agonists for each P2 receptor subtype [19,20].

P2X7R antagonists can be divided into at least six major groups. The first group comprises divalent cations, such as Ca^2+^, Mg^2+^, Zn^2+^, Cu^2+^, and H^+^. These cations inhibit the ATP-induced activation of P2X7Rs, showing IC_50_ values *in vitro* (in µM) of 2900, 500, 11, 0.5, and 0.4 (e.g., pH 6.1) respectively, acidification of pH modifies the charge on histidine residues, inhibiting the ATP-gated current [21,22,23,24]. The second group comprises the general P2R antagonists, such as PPADS (pyridoxalphosphate-6-azophenyl-2′,4′-disulfonic acid) and suramin, with lower potencies compared with other P2 receptors and lower selectivity for P2X7R. The ionic currents generated upon ATP application are relatively insensitive to blockage by suramin (IC_50_ > 300 µM for rat P2X7R (rP2X7R), which might also interfere with other pathways [25], and PPADS (IC_50_ ~10 µM) [24]. In addition, other P2R antagonists are moderately selective for P2X7R and have either low (oxidized ATP, oxATP) or high (Brilliant Blue G, BBG) potency. For example, BBG blocks rP2X7 at a concentration of 10 nM and human P2X7R (hP2X7R) at a concentration of 200 nM [26,27]. BBG also blocks voltage-gated sodium channels and P2X4R at low micromolar concentrations [28]. oxATP acts as a P2X7R irreversible antagonist at 100–300 µM, after only a 1–2 h incubation [29,30,31,32].

The third group comprises the large organic cations, calmidazolium and KN-62. Calmidazolium (10 nM) blocks rP2X7R currents in an easily reversible and voltage-independent manner [22] but is less effective for the activation of hP2X7R [33]. The piperazine-derived antagonist KN-62 blocks calcium-calmodulin-dependent protein kinase type II (CaM kinase II). Ionic currents are inhibited in cells expressing hP2X7R or mP2X7R that have been treated with nano- and micromolar ranges of KN-62, respectively, although this antagonist exhibits little effect on rP2X7R [34].

The fourth group comprises the compounds derived from natural products. For example, benzofenatridine alkaloids, such as chelerythrine, and mineral oil inhibit P2X7R function [35,36], although castor oil may activate P2X7R [37]. Emodin (1,3,8-trihydroxy-6-methylanthraquinone) reduces the P2X7R activity in both macrophages and rP2X7R-expressing HEK-293 cells [38]. In 2011, our group observed that *Rheedia longifólia* extract inhibits the P2X7R activation. Using chromatography, we showed that the most active compound identified was the bisflavanoid amentoflavone [39]. Recently, Marques da Silva and collaborators demonstrated that colchicine inhibits P2X7R-induced pore formation, but does not affect the P2X7R cationic function [40]. This inhibition may be related to microtubule inhibition with consequent impairment of cationic P2X7R dilation or other possible mechanisms. Another possibility is the restriction of P2X7R C-terminus movement, once it is associated with P2X7R pore formation. For a more detailed description of P2R and natural products, see Faria *et al.* [41] and Soares-Bezerra *et al.* [42]. 

The fifth group comprises monoclonal antibodies, which act against different regions of the P2X7R and are capable of blocking current and dye permeability [43,44].

The sixth group comprises new synthetic molecules, which enclose a variety of structural and chemical conformation types. Many pharmaceutical companies, including Pfizer [45], GlaxoSmithKline [46,47], and Abbott Laboratories [18,48], have discovered new synthetic molecules and described their activity on P2X7R. 

These novel molecules are in pre-clinical or clinical trial phases, show promising results, and are leading to patents. The details for some compounds and their activities towards P2X7R have been previously published, while the details for other compounds remain unpublished. For more detailed information about these compounds, we suggest the following reviews: Friedle *et al.* [49]; Gunosewoyo *et al.* [50]; and North and Jarvis [51]. 

The cyanoguanidine derivative A-740003, developed by Abbott Laboratories, is one of the compounds that is in the pre-clinical or clinical trial phase and whose results are published [52]. Nanomolar concentrations of A-740003 inhibit P2X7R function for both rat and human receptors, and the ionic channel and pore functions were inhibited in human THP-1 cells. In addition, A-740003 inhibits behavioral responses in rat models of mechanical allodynia and thermal hyperalgesia [52]. These animals also were treated with a di-substituted cyanoguanidine derivative, A-438079, which was more potent and selective than A-740003 for the inhibition of hP2X7R and rP2X7R as opposed to mP2X7R, which is less sensitive than the other mentioned channels species [19]. In 2009, Abbott Laboratories discovered the competitive aminoquinoline-derived inhibitor A804598, which exhibits greater potency and selectivity than A-740003 or A-438079 on human, rat, and mouse P2X7R [25].

In the same year, AstraZeneca Laboratories produced a selective and potent cyclic imide (AZ11645373) that inhibits hP2X7R at low nanomolar concentrations [53]. However, the action of this amide is slowly reversed, and is much less effective in rats. The P2X7R antagonist *N*-[2-[[2-[(2-hydroxyethyl)amino]ethyl]amino]-5-quinolinyl]-2-tricyclo[3.3.1.13,7]dec-1-ylacetamide dihydro-chloride (AZ10606120) inhibited P2X7R activation in electrophysiology experiments. Under conditions that activate the cationic channel and the pore, this antagonist reduced BzATP-induced ionic currents at low micromolar and nanomolar concentrations in humans and rats, respectively [54]. Moreover, 3-[1-[[(3-nitro[1,1-biphenyl]-4-yl) oxy] methyl]-3-(4-pyridinyl)propyl]2,4-thiazolidinedione (AZ11645373) is a selective and potent (IC_50_ 5–10 nM) allosteric hP2X7R antagonist that exhibits species selectivity because rP2X7R is not affected [53].

GlaxoSmithKline also developed an adamantine derivative, *GSK314181A*, that inhibits hP2X7R functions at moderate nanomolar concentrations and inhibits rat receptors at micromolar concentrations [55]. This compound reduces carrageenan-induced acute pain and inhibits P2X7-associated pore formation and IL-1β release in a competitive manner in human THP-1 cells.

Pfizer, Abbott, Affectis Pharmaceuticals AG, AstraZeneca, Evotec, and Merck, among other pharmaceutical groups have developed and patented even more compounds with currently unpublished functional properties. Some studies are in the initial phase, while other studies have presented partial results, as detailed in a review published in 2010 [56].

Although a number of new P2X7R antagonists have been developed, the majority of these compounds are selective for P2X7R (P2X7R pharmacological groups 1 to 4) or exhibit low pharmacokinetic activity in humans [56,57]. Consistently, researchers have attempted to develop new P2X7R antagonists based on natural or synthetic molecules, including more precise studies concerning the pharmacokinetics of human P2X7R and the actions of these compounds on the profile of other cytokines released during inflammation, e.g., TNF-α.

## 4. The Role of P2X7R in Inflammation

ATP and its related extracellular nucleotides induce a wide variety of pathophysiological responses during the inflammatory response through the activation of P2 purinergic receptors that are present on the cell surface. The pathophysiological role of purinergic signaling occurs through the activation of different pathways that may include calcium mobilization, actin polymerization, chemotaxis, mediator release, cell maturation, cytotoxicity, and cell death [58]. The inflammatory process is driven through the stimulation of cells of the immune system, which secrete cytokines that play an essential role in both the development and maintenance of the inflammatory reaction. The release of a major proinflammatory cytokine, IL-1β, from monocytes or macrophages is associated with P2X7R activity [59]. Following the activation of P2X7R through high concentrations of and/or prolonged exposure to ATP, a large conductance channel is elicited, which leads to dynamic changes in the membrane potential that include an intracellular potassium efflux. This cationic efflux induces inflammasome complex assembly and subsequent pro-caspase-1 maturation into caspase-1 through the NOD-like receptor protein (NLRP3). Caspase-1 plays a key role in the cleavage of pro-IL-1β to form mature IL-1β, which plays a principal role in nitric oxide synthase (NOS), cyclooxygenase-2, and tumor necrosis factor-alpha (TNF-α) activities [59]. Similarly, other studies have described the relationship between P2X7R activation and the release and activation of other cytokines, such as IL-1α [60], IL-2 [61], IL-4, IL-6, IL-13, IL-18 [58,59,60,61,62], and TNF-α [59], and inflammatory mediators, such as nitric oxide (NO) [57] and superoxide anions [63,64]. However, other signaling pathways have been associated with P2X7R activation, including phospholipase D (PLD), nuclear factor kappa B (NF-κB), and mitogen-activated protein kinases (MAPKs) [59]. Several *in vivo* animal models have demonstrated the proinflammatory effect of P2X7R through the release of IL-1β [64,65,66,67,68,69]; other cytokines [70,71], and inflammatory mediators [70,72]. However, the selectivity of these molecules for P2X7R has not been investigated. 

The release of increased amounts of intracellular ATP during inflammation increases paracrine purinergic signaling, thereby activating P2X7R, which acts as a “find-me” signal for apoptotic cell clearance *in vivo* [73]. The activation of P2X7R in this event promotes the formation of large conductance channels, which are associated with subsequent apoptosis, following this activation. Moreover, reactive oxygen species (ROS) are also generated during death-associated events [74].

ROS act as important mediators for c-Jun N-terminal kinase (JNK 1 and JNK) activation and interact with nucleotide receptor-mediated p38 [74]. ROS formation leads to the activation of NF-κB, MAPKs, extracellular signal-regulated kinases (ERK 1/ERK 2), p38, JNK 1/JNK 2 [74], and inflammasome complex assembly [24]. Moreover, the regulation and expression of these pathways is mediated through the C-terminal domain of P2X7R, which is considered a key signaling site, with respect to P2X7R-associated pore formation [75]. Marques da Silva and colleagues (2011) showed that colchicine treatment inhibits P2X7R pore formation *in vitro* [40]. Treating mice with LPS and ATP induces ROS, NO, interferon-γ, and IL-1β production, and colchicine treatment inhibits this response [40]. The *Glu496Ala* loss-of-function polymorphism in P2X7R diminishes pore formation, cytokine release, and cytotoxic effects in an *ex vivo* whole blood model [76]. Because this polymorphism does not affect the activity of the intrinsic low conductance cationic channel, pore formation is associated with proinflammatory cytokine release and cell death. These results suggest the potential dependency between P2X7R pore formation and cytokine release. In addition, a few studies have shown an association between pore formation and P2X7R function, perhaps, reflecting the low selectivity or low pharmacokinetic and pharmacodynamic activity in animal models [56,57,58].

The inflammatory disease rheumatoid arthritis has become a target for the applicability of the P2X7R, which has gained considerable attention worldwide [25]. With 1.9% worldwide prevalence, rheumatoid arthritis is a chronic inflammatory condition that can lead to a permanent incapacitating functional state [77]. The primary cells that are involved in cartilage damage, including fibroblasts, macrophages, and activated lymphocytes, express purinergic receptors, including P2X7R. These receptors are over-activated in response to the increased release of either extracellular nucleotides or nucleosides, as evidenced by the identification of ATP in the synovial fluid of patients with rheumatoid arthritis [77]. As previously described, P2X7R activation triggers the production and release of proinflammatory cytokines, such as IL-6, IL-18, and TNF-α, which induce and perpetuate the inflammatory process. IL-1β release is primarily associated with macrophages, which are present in the synovial fluid, and the release of this cytokine promotes lymphocyte infiltration, leading to the synovial hyperplasia and the inhibition of cartilage synthesis [78,79,80].

Rheumatoid arthritis is mediated through P2X7R activation in animal models [78], and immune cells from human arthritis patients show augmented function resulting from the His155Tyr (489C > T) single nucleotide polymorphism P2RX7 [81,82]. In contrast, pre-clinical and clinical trials with the P2X7R antagonist in patients with rheumatoid arthritis failed to inhibit disease progression [83,84]. This inefficient P2X7R antagonist activity requires the development of new compounds or the enhancement of pharmacokinetic or pharmacodynamic ameliorating effects of the existing molecules. 

P2X7R mediates neuropathic pain that is caused by the abnormal activation of the nociceptive pathway at the spinothalamic stage [85]. ATP is an important mediator in the co-transmission of sensory information from the peripheral to the central nervous system, and P2X7R plays a key role in neuropathic pain [86].

Peripheral neuronal cell damage modulates nociceptive pathways through the release of many algogenic compounds, such as ATP, which activate P2X7R-expressing microglial cells. Following P2X7R activation, proinflammatory cytokines, such as IL-1β, are released, and superoxide anions, such as hydrogen peroxide, are generated and maintain the pain [77]. 

Based on these results, multiple scientific groups have suggested P2X7R as a therapeutic target for pain management.

Moreover, in addition to promoting pain and inflammation, P27XR also induce chronic conditions. Excessive activation of the P2X7R stimulates cytoskeletal reorganization and, consequently, membrane pore opening, which eventually lead to immune cell death [77]. 

## 5. The Roles of P2X7 Receptors in Pain Disorders

The physiological role of pain is to highlight potential threats to the welfare and integrity of the body and maintain attention until the cause of pain activation has been identified and removed [87]. The warning function of pain reflects the activation of nociceptors, which are sensitized when the stimulus is potentially dangerous, *i.e.*, above a certain physiological range or more than an innocuous stimuli [88,89]. Nociception is defined as the perception of pain, and this phenomenon is driven through the peripheral nerves. Nociceptors are widely distributed in the skin, blood vessels, muscles, joints, and organs and are sensitive to thermal, mechanical, and chemical stimuli. Among the nerve fiber subtypes, Aδ and C are the most responsible for driving the sharp/quick and diffuse/long-lasting pain stimuli, respectively [90]. The stimulation of peripheral nociceptors causes nociceptive information to be gathered through afferent fibers and transmitted to the central nervous system. The long-fiber nociceptive axons, which are located in the peripheral nerves, extend from their cell bodies into the dorsal root ganglion. Extending from the cell body, the axon bifurcates primary afferent extensions to simultaneously send signals to the spinal cord and tissues of the body [91]. These nerves have a large number of different nociceptor families, such as transient receptor potential channels, sodium channels Nav1.5 and Nav1.7, and P2 receptors. These receptors act at different stages during the pain stimulus, such as the transmission of signal transduction and modulation; however, these channels are not limited to a single characteristic [91].

The effect of ATP on pain has been studied since the 1960s. These seminal studies showed that ATP provokes persistent pain and exhibits nociceptive action when applied to human skin blisters and in mice [92]. Consistent with Collier’s work, Bleehen and colleagues demonstrated that the major algogenic compounds identified in the lysates of human erythrocytes applied onto skin blisters were ATP and its derivatives [93]. In 1972, Burnstock *et al.* formulated the purinergic neurotransmission hypothesis, and since then, many studies have demonstrated that both neurons and glia release ATP, which acts on purinergic receptors in glial cells [94]. These results showed the critical roles of extracellular ATP and the promising development of this molecule as an analgesic compound, thereby providing a better understanding of pain mechanisms.

Thus, many studies focused on purine and pyrimidine molecules in the context of neurotransmission have demonstrated the distinct roles of the purinergic receptors in the nervous system [95,96]. Furthermore, the activities of purinergic receptors have also been demonstrated in neurons, glial cells, and leukocytes; thus, the display and modulation of pain-related stimuli also contribute to inflammatory and neuropathic pain [77].

As previously discussed, activation of P2X7R may promote inflammation and cell death. Despite the characterization of how this receptor participates in the pain process, it remains unclear how P2X7R is involved in the spinal cord environment or whether P2X7R is included in another context beyond the microglia [97]. Moreover, following the activation of the ionotropic purinergic receptors, such as P2X7R, the intracellular concentration of calcium, an important second messenger, is increased; calcium modulates neuronal excitability in P2X7R-expressing glial cells [98,99]. In addition, the glutamate release mediated by P2X7R activation in neurons [100] may be an essential component to increase the excitability. These factors suggest that P2X7R is a key element in the development of pain. In the following sections, two distinct types of pain will be discussed concerning the roles and protective actions of P2X7R in these types of pain.

### 5.1. Inflammatory Pain

Many different types of substances have been used in inflammatory pain assays, depending on the animal models. Formalin, complete Freund’s adjuvant (CFA), and carrageenans are most commonly used agents, which reflects their ability to evoke either the sensitization or activation of the immune system to alter the algogenic molecules (immune mediators) [101,102]. 

Beyond the participation of P2X7R in chronic and neuropathic pain, Itoh *et al.* [103] showed that the P2X7R blockers BBG and oxATP successfully attenuate the effects of inflammatory pain elicited with the irritant transient receptor potential ankyrin 1 agonist mustard oil. Moreover, the selective and potent P2X7R agonist BzATP activates medullary dorsal horn nociceptive neurons with a 3-fold greater potency than ATP. Nevertheless, not all aspects of the mustard oil-induced acute pain assay are blocked by these two P2X7R antagonists, suggesting the putative contribution of other P2 receptors. The acute inflammatory pain model has been important for associating the functions of P2X7R with this pain subtype in the microglial context. The results have shown that these compounds have similar effects as the microglial blocker minocycline, which has neuroprotective actions and attenuates the activated microglial responsiveness in chronic pain [104,105,106]. Interestingly, pyridoxal-phosphate-6-azophenyl-2,4-disulfonic acid, a non-selective purinergic, also blocks MO-induced central sensitization in the medullary dorsal horn, via a different mechanism than by the P2X1, P2X3, and P2X2/3 receptor antagonist 2,3-O-2,4,6-trinitrophenyl-ATP (TNP-ATP), which only partially inhibits this sensitization [106].

One of the principal causes of pain in arthritis is inflammation in the joints. The P2X7R antagonist (oxATP), promotes pain relief in Wistar rats with arthritic paws, following the administration of complete Freund’s adjuvant (CFA), including a reduction of edema in the inflamed paw [107].

In addition, inflammation involves the activation of P2X7R. Inflammasome assembly might also be required for inflammation in LPS-primed monocyte-derived cells, for example, to promote the release of interleukin-1β. P2X7R has also been associated with the release of PGE2 via MAPK activation to maintain a chronic inflammatory response [58]. Several authors [108,109,110] have suggested that once the binding site is blocked, oxATP inhibits P2X7R activity and consequently impairs cytokine release [108].

As the P2X7R plays a role in the immune response, such as the activation of some cytokines, the inhibition of this receptor by antagonistic molecules could be useful in the treatment of some autoimmune diseases, or through the inhibition of a chronic inflammatory response that entails degenerative processes, *i.e.*, such as cartilage destruction in arthritis [58,77]. Furthermore, the blockage of the P2X7 and neuroprotection in the Alzheimer’s disease context is also related [111]. Indeed, it was also demonstrated that the P2X7R plays a role in the CNS, which leads to a neuroprotection through the release of IL-1β and the upregulation of P2Y2 [112]. Taken together, these findings provide evidence of a protective pathway related to the antagonism of this receptor.

### 5.2. Neuropathic Pain

The International Association for the Study of Pain attributes pain responses to lesions or diseases in the somatosensory nervous system [113]. This pain type might also reflect nerve section or nerve compression in neuropathic pain assays.

To evaluate the roles of P2X7R in neuropathic pain, another P2X7R antagonist, A-740003, was identified as an important component in the neuropathic pain context. Honore and colleagues [52] performed two distinct assays in which both chronic constriction damage of the sciatic nerve and vincristine-induced neuropathic effects were attenuated when treated with A-740003. In addition to inhibiting channel properties *in vitro*, as evidenced by changes in intracellular calcium concentration, interleukin-1β release, and pore dilation, A-740003 also inhibited tactile allodynia *in vivo* [52]. Allodynia is defined as pain following a stimulus that does not normally provoke pain.

Similar results were observed with another P2X7R antagonist, A-438079. This compound blocked allodynia in all three neuropathic pain assessments: chronic constriction injury, ligation of spinal nerves, and vincristine-induced neuropathy. In non-neuronal cells, A-438079 also affected interleukin-1β release, suggesting a key role for P2X7R in neuro-inflammatory processes [114]. 

Consistently, Chessell and colleagues (2005) [65] showed that the absence of P2X7R disrupts both inflammatory and neuropathic pain after mechanical or thermal stimuli. The release of interleukin-1β is also impaired in this context, demonstrating a functional role of this receptor in these pathological processes. Interestingly, this group also showed that P2X7R expression is up-regulated in human dorsal root ganglia and the injured nerves of patients affected with neuropathic pain [65]. 

Zhang *et al.* [115] observed no anti-P2X7R immunoreactivity in rat dorsal root ganglia neurons, whereas this receptor was expressed in non-neuronal cells. These authors performed an electrophysiology assay, in which DRG neurons exposed to 100 µM BzATP showed rapid P2X3 receptor (P2X3R) expression and a desensitized transient current, which the P2X3R selective antagonist A-317491 successfully inhibited. This response showed that P2X3R is the primary purinergic receptor in neurons whereas in non-neuronal cells, A-317491 was unable to inhibit the activity of BzATP. Moreover, a pharmacological profile similar to that of rat P2X7R in peritoneal macrophages, with a rank order of BzATP > ATP > αβ-meATP, was observed [116]. Nevertheless, one of the P2X7R hallmarks, non-selective pore formation, was not observed when whole-cell voltage clamp currents were abolished after the Na^+^ extracellular solution was changed to an *N*-methyl-D-glucamine (NMDG^+^) bath solution after prolonged exposure to the selective agonist BzATP [116]. Other studies using distinct strategies have shown that P2X7R-induced pore formation is functionally associated with mechanical allodynia in both mice and humans and chronic pain in humans [117]. The P2X7R pore formation may be used as a new strategy for the treatment of some cases of chronic pain.

Studies using P2X7R KO mice have revealed variability in the pain response because the potential for contamination with splice variants is likely [118]. Using a mouse P2X7R(-/-) model, Barberà-Cremades *et al.* [119] observed a decrease in the febrile response evoked by LPS or IL-1 β, demonstrating the importance of this receptor on the development of inflammatory disorders (Table 1). Consistently, Chessell *et al.* [65] demonstrated that the P2X7R(-/-) mouse model is insensitive to both neuropathic and inflammatory pain stimuli. Although Tsuda *et al.* [120] showed that P2X4R(-/-) mice had an impaired response to inflammatory and neuropathic pain stimuli, this profile was not reproducible in acute pain or tissue damage-induced pain, suggesting that P2X4R participates in chronic inflammatory and neuropathic pain. Interestingly, Hansen *et al.* [118] reported the lack of an inhibitory response in P2X7R(-/-) mice in the bone-pain context, which is different from the inflammatory or neuropathic pain responses. These authors suggested the existence of mice with variants of P2X7R, which might be responsible for this result.

**Table 1:** Pain disorder responses in P2X7R KO mice models.

Pain Model	Knockout Used	Experimental Context	Observed Effect	Reference
Inflammatory	P2X7R(-/-)	Fever by LPS or IL-1β intraperitoneal inoculation	Decreased febrile response in knockout mice	[119]
Bone cancer pain	P2X7R(-/-)	Inoculation with 4T1 mammary cancer cells or NCTC 2472 osteosarcoma cells	No defects in bone cancer pain, probably related to P2X7 receptor splice variants expressed in the knockout mice	[118]
Inflammatory and neuropathic	P2X7R(-/-)	Inflammatory (in an adjuvant-induced model) and neuropathic (in a partial nerve ligation model	Abolished pain sensitivity in both pain models	[65]

## 6. P2X7 Receptor as a Novel Therapeutic Target

The first medicine based on P2 antagonism was ticlopidine, which binds to the P2Y12 receptor on platelets, reducing activation and aggregation [121]. Currently, these antagonists are in the third generation (e.g., prasugrel) and have reduced acute coronary syndrome-associated death. With regard to P2X receptors, there is no medicine (agonists or antagonists) in clinical use. However, pre-clinical studies indicate that P2X7R antagonists might be used to treat inflammatory or partially inflammatory diseases in any tissue, such as chronic pulmonary conditions, glomerulonephritis, rheumatoid arthritis, inflammatory bowel diseases, stroke, brain trauma, amyotrophic lateral sclerosis, multiple sclerosis, and pain disorders. Therefore, P2X7R has become a pharmacological target for several pharmaceutical companies, with several clinical trials in phase I and II to evaluate the efficacy of P2X7R antagonists on many diseases (Table 2). Nevertheless, the clinical trials do not address the use of a high conductance channel associated with P2X7R to increase the passage of hydrophilic drugs to the cytoplasm of cells expressing the P2X7R pore as a drug delivery method. 

A potential target for the use of P2X7 agonists involves neoplastic cells that express high levels of P2X7 receptors, which facilitate the passage of hydrophilic chemotherapeutic agents. Results from our group (yet unpublished) have shown that ATP and hydrophilic chemotherapy drugs synergistically induce cellular death in tumor cells expressing P2X7 receptors. The induction of apoptosis through the P2X7R, emerges as a promising therapeutic target focusing the cancer treatment [122].

Other diseases in which ATP agonists might exhibit therapeutic potential include tuberculosis (TB), a chronic infectious condition that is caused by the facultative intracellular pathogen *Mycobacterium tuberculosis* (MTB). This disease is a global health problem that affects one-third of the world population. Among infected individuals, approximately 5%–10% of these persons will develop the clinical disease. Macrophages act as the major host cells for intracellular *Mycobacterium* replication, and these cells also regulate the growth and viability of this pathogen. Several groups have shown that P2X7R activation induces apoptosis and autophagy in infected macrophages, leading to the *Mycobacterium* death. In addition, several polymorphisms have been associated with TB in different ethnic groups. Thus, ATP agonists might be associated with common antibiotics used to treat tuberculosis [123,124,125].

**Table 2:** Recently evaluated compounds with promising antagonistic activity against P2X7R.

Compound	Company	Study	Trial Phase	Completed	Observed Result	Observed Side Effect	Reference
A-438079	Abbott Laboratories	Neuropathic and inflammatory pain	Pre-clinical	Yes	Inhibited mechanical allodynia and effective in the formalin pain model	Not evaluated	[114]
A-740003	Abbott Laboratories	Neuropathic and inflammatory pain	Pre-clinical	Yes	Analgesic effect in inflammatory and neuropathic pain in rat	Not evaluated	[52]
A804598	Abbott Laboratories	Neuropathic and inflammatory pain	Pre-clinical	Yes	Analgesic effect in inflammatory and neuropathic pain in rat	Not evaluated	[25]
A847227	Abbott Laboratories	Anti-inflammatory and neuropathic pain	Pre-clinical	Yes	Antiallodynic activity and reduced the release of IL-1β in the zymosan mouse model	Not evaluated	[55]
AZ10606120	AstraZeneca	Ligand interaction and binding with P2X7R	Pre-clinical	Yes	Its molecule binds to a site different from which ATP binds, but it acts as a negative allosteric modulator	Not evaluated	[25]
AZ11645373	AstraZeneca	Selective potency	Pre-clinical	Yes	Inhibited hP2X7R and its pharmacologic properties	Not evaluated	[53]
AZD9056	AstraZeneca	Rheumatoid arthritis	II	Yes	No efficacy	Gastrointestinal (nausea, diarrhea, and vomiting)	[84]
CE-224,835	Pfizer	Rheumatoid arthritis	II	Yes	No efficacy	Gastrointestinal (nausea and diarrhea)	[83]
EVT-401	Evotec	The pharmacokinetic profile and pharmacodynamic effects	I	Yes	Safe and well tolerated	Not related	[78]
GSK314118A	Glaxo	Inflammatory pain	Pre-clinical	Yes	Analgesic activity in rat CFA model of inflammatory hyperalgesia	Not related	[55]

## 7. Conclusions

Although inflammatory diseases afflict many people worldwide, the current treatments for these diseases have undesirable side effects. Research in this area has attempted to develop new therapies and medicines to efficiently treat these diseases while offering fewer undesirable effects to the patient. In this context, P2X7R emerges as a promising target to treat inflammatory conditions because this receptor is involved in the release of proinflammatory cytokines that play an important role in the development of inflammation and pain. Thus, molecules that modulate the physiological functions associated with the activation of this receptor, acting as an antagonist, could be used to develop a new class of anti-inflammatory medicines, with future clinical applicability. 
